# Transmesenteric Internal Herniation Leading to Small Bowel Obstruction Postlaparoscopic Radical Nephrectomy

**DOI:** 10.1155/2017/5128246

**Published:** 2017-03-30

**Authors:** G. A. Cuthbert, L. T. Teo

**Affiliations:** Tan Tock Seng Hospital, 11 Jalan Tan Tock Seng, Singapore 308433

## Abstract

Internal herniation following laparoscopic surgery is rare. We present a case of small bowel obstruction secondary to internal herniation in a 76-year-old male patient. Presentation was on postoperative day 28 following transperitoneal laparoscopic radical left nephrectomy for suspected renal carcinoma. The herniation was through a defect in the large bowel mesentery identified at exploratory laparotomy. To date, 10 cases of internal herniation following laparoscopic nephrectomy have been described in the literature. Two cases were managed laparoscopically and the remainder by laparotomy. One case required resection of an ischaemic portion small bowel and the remainder were managed by reduction of the hernia and closure of the defect. Internal herniation is rare but carries significant morbidity. It must be considered in cases presenting with obstructive symptoms after laparoscopic nephrectomy. Early CT scanning and prompt surgical management are hallmarks of best management.

## 1. Introduction

A 76-year-old Chinese gentleman presented to our surgical department with acute central and lower abdominal pain. He was at postoperative day 28 following laparoscopic radical left nephrectomy performed via a transperitoneal approach. A diagnosis of small bowel obstruction was made based on computed tomography (CT). Findings included dilated small bowel loops and transition points at both the distal ileum and duodenojejunal flexure. An exploratory laparotomy was performed after a failed trial of conservative management. Intraoperative findings were small bowel obstruction secondary to internal herniation of the small bowel through a defect in the large bowel mesentery. Small bowel resection and primary anastomosis were performed followed by repair of the mesenteric defect. The patient was discharged from hospital on postoperative day 13 following resolution of postoperative small bowel ileus.

## 2. Case Report

A 76-year-old Chinese male presented to the emergency department with a 12-hour history of acute central and lower abdominal pain which was constant and colicky in nature. He denied any nausea or vomiting; he had opened his bowels normally that morning and was still passing flatus. He was noted to be at postoperative day 28 following laparoscopic radical left nephrectomy. This was performed for a mass found in the upper pole of the left kidney and was completed via a transperitoneal approach.

He is a lifetime nonsmoker and denies regular alcohol consumption. He is fully independent and still works as a taxi driver. His past medical history includes a D1 duodenal ulcer with a recent admission for upper gastrointestinal bleeding. He also takes regular medication for hypertension, hyperlipidaemia, benign prostatic hypertrophy, and gout.

On presentation to the emergency department he was afebrile and had a blood pressure of 156/69 and a heart rate of 87 beats per minute. On examination he had a soft abdomen with central tenderness and no guarding. Hernial orifices were normal and on digital rectal examination the rectum was empty and no masses were palpable.

**Figure 1:**
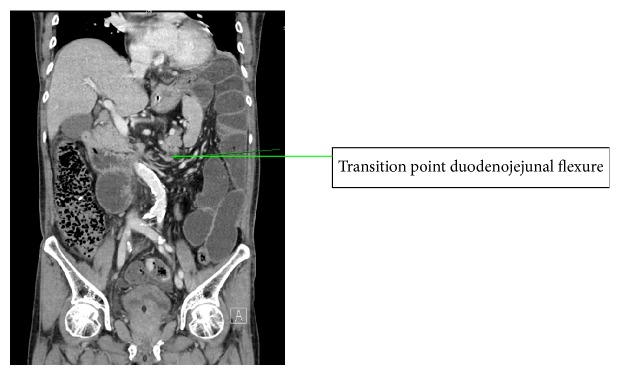
Coronal slice of CT showing duodenojejunal flexure transition point in the left hemiabdomen.

Initial abdominal X-ray showed no dilated bowel loops and the working diagnosis was adhesion colic in view of his recent operation. Within 24 hours of admission a contrast enhanced CT scan was performed in view of clinical deterioration, rising lactate, and worsening metabolic acidosis and acute kidney injury. At CT the findings were small bowel obstruction, suggestion of 2 transition points in the left hemiabdomen (distal ileum and duodenojejunal flexure), and no evidence of ischaemia (Figure 1).

An emergency exploratory laparotomy was performed after review of the CT findings. Findings at laparotomy included almost complete small bowel herniation up to distal ileum through a descending colon mesenteric window. There were 2 transition points. The first was at the distal ileum at the point of herniation; the second was 40 cm from the duodenojejunal flexure where the jejunum was adherent to the retroperitoneum at the site of the left nephrectomy pedicle. There were omental adhesions noted to mesh in the right inguinal region consistent with previous laparoscopic transabdominal preperitoneal inguinal hernia repair. The small bowel was distended but healthy and the colon was healthy.

The operative procedure consisted of reduction of the small bowel through the mesenteric window and division of jejunal-retroperitoneal adhesions. An inadvertent enterotomy was made at the adhesion site causing some peritoneal soiling. The small bowel was decompressed through the enterotomy site and after resection of a short segment of jejunum a functional end-to-end stapled anastomosis was made.

**Figure 2:**
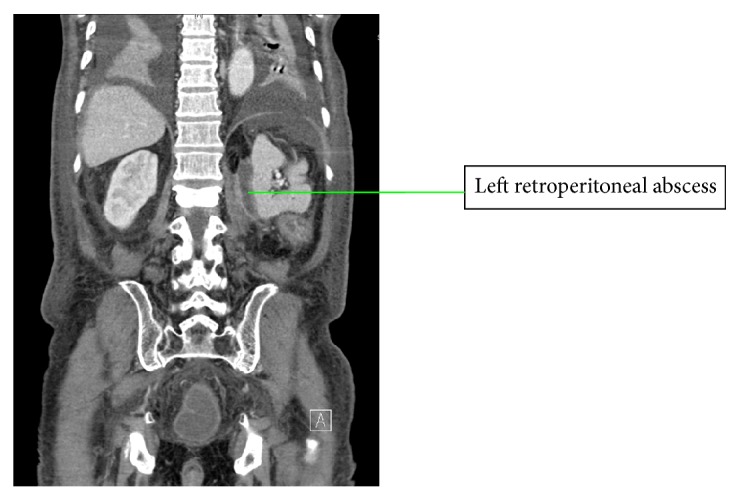
Coronal slice of CT showing postoperative left retroperitoneal abscess.

Postoperatively the patient was treated with intravenous antibiotics for a presumed aspiration pneumonia thought to have been caused by vomiting during induction of anaesthesia. He was managed for postoperative ileus which resolved on postoperative day 4 and was discharged from hospital on postoperative day 12. The patient was readmitted on postoperative day 25 and managed conservatively for ileus. CT of the abdomen and pelvis on admission showed a 2.3 × 3.6 × 6.6 cm abscess in the left retroperitoneal region abutting the psoas muscle (Figure 2). The abscess was treated with intravenous antibiotics and percutaneous drainage withheld as clinically the patient improved and ileus resolved. He was discharged on postadmission day 7. Interval CT of abdomen and pelvis shows reduction in the size of the retroperitoneal abscess.

## 3. Discussion

Laparoscopic radical nephrectomy has been adopted as the preferred surgical approach in cases of renal cell carcinoma. It has been shown to provide equivalent cancer control and lower complication rates when compared with an open approach [1, 2].

A retrospective analysis of over 7000 patients undergoing open or laparoscopic radical nephrectomy by Tan et al. showed that the laparoscopic approach offered the benefit of lower complication rates when compared with an open approach. Mortality and failure-to-rescue rates in patient with complications after having laparoscopic surgery were significantly higher however when compared to patients undergoing open surgery [3]. This study also identified that in mortality cases gastrointestinal and iatrogenic injuries were almost twice as common in the laparoscopy group. The authors suggest that this finding may be attributed to challenges in recognition and management of bowel and vascular injuries and the substantial learning curve associated with laparoscopic nephrectomy.

Bowel complications are rare in laparoscopic nephrectomy, accounting for 1% of total complications [4], being most commonly postoperative ileus. Our case describes internal herniation and intestinal obstruction.

A literature search identified 8 publications reporting a total of 10 cases of internal herniation following laparoscopic nephrectomy. Five were carried out for suspected or confirmed cancer and the remaining 5 donor nephrectomies. All 8 cases presented with small bowel obstruction and required operative management. One case progressed to small bowel gangrene and required small bowel resection. The remaining cases were managed by reduction of the hernia and closure of the defect. All 10 cases survived their hospital admission and were discharged from hospital.

All 10 cases described the internal herniation resulting from a tear in the colonic mesentery. In all cases the mesenteric defect was made inadvertently and not as an intended step in the operation. The defect is made when an incision is made in the lateral colonic mesentery in order to mobilise the colon medially and access the kidney [5]. Creation of such defects was first reported in cases of donor nephrectomy. It has been attributed to extensive colonic mobilisation and mesenteric dissection in order to maximise renal vessel length available for vascular anastomosis [4]. The case we present here is of radical nephrectomy for suspected cancer. The dissection in radical nephrectomy differs from donor nephrectomy as it prioritises cancer-free margins. A number of authors attribute the propensity for internal herniation in radical cases to the large potential space created during wide preparation of the major vessels and radical lymphadenectomy [4, 6]. Kumar et al. propose 4 factors aiding the formation of internal herniation in cases of left nephrectomy; the attachment of the small bowel mesentery favours disposition to the left iliac fossa under gravity, as the left colon is in the way, a defect forms a passage of least resistance, the potential space created by the nephrectomy, and the ligament of Treitz creates a pivot point for rotation of the small bowel towards the left of the abdomen [7].

Six cases identified from our literature search used CT to diagnose internal herniation. A CT scan in 2 of these cases identified a mesenteric defect and evidence of internal herniation. In the remaining 4 cases small bowel obstruction was reported but the diagnosis of internal herniation was not made until laparotomy. CT findings in these cases include dilated small bowel, evidence of small bowel venous congestion, pneumatosis coli, and translocation of the small bowel loops to the respective renal fossa. In 2 cases the patient underwent exploratory surgery following laboratory investigations and plain X-rays only. In one case the mesenteric defect had been identified during laparoscopic nephrectomy. In this case prior knowledge of the presence of such a defect may have negated the need for CT. This patient underwent exploration laparoscopy and laparoscopic repair of the defect after reduction of the hernia. In a different case Kumar et al. proceed straight to exploratory laparotomy without performing a CT scan. It is unclear why CT scanning was not used in this case. In the 2 remaining cases reported in the literature preoperative imaging is not discussed.

Of the cases described 1 was managed laparoscopically, 1 was laparoscopically assisted, and the remainder was managed by laparotomy. In the case describing laparoscopically assisted exploration the mesenteric defect was recognised during laparoscopic nephrectomy but was not closed [8]. The surgeon intentionally enlarged the orifice in order to prevent bowel obstruction occurring postoperatively. The awareness of the mesenteric defect in this case may have made laparoscopic exploration more feasible. It is unclear however whether or not this case was converted into open surgery. The case described by Wadhawan et al. is managed entirely laparoscopically [9]. In this case the hernia is reduced following dissection of the small bowel from the tail of the pancreas. The defect is then sutured closed.

In only one case small bowel resection was performed [5]. In this case the authors performed an exploratory laparotomy and resected 150 cm of gangrenous small bowel. This case was 9 weeks after laparoscopic radical nephrectomy. The authors advocate that following laparoscopic nephrectomy the mesentery should be inspected and any defects repaired. The authors recommend that internal herniation should be considered as a possible cause of bowel obstruction postlaparoscopic nephrectomy and laparoscopy performed.

In all reported cases the mesenteric defect was closed after identification and reduction of the internal hernia.

When an inadvertent or intentional mesenteric defect is recognised during the primary operation the surgeon can choose to leave the defect behind, repair it, or extend it. Traditionally we teach that hernias with a narrow neck are more likely to be incarcerated and become complicated compared to those with a wide neck. Although wide necked hernias are potentially uncomfortable or unsightly, they are less likely to be incarcerated as contents move freely in and out of the sac. In the case described by Regan et al., the mesenteric defect was recognised at primary operation [8]. The defect could not be closed laparoscopically and was therefore extended to reduce the risk of incarceration in the event of internal herniation. In this particular case the patient developed small bowel obstruction within the first postoperative week and underwent laparoscopic reduction of the hernia repair of the defect. Wong et al. suggest that although large defects are thought to be less likely to cause obstruction, adhesions around the defect may contribute to a higher likelihood of incarceration in the immediately postoperative patient. Wong et al. further mention that postoperative intestinal obstruction from internal herniation should take on a delayed presentation compared with bowel injury or ileus. In cases of postoperative ileus or bowel injury presentation should be in the immediate postoperative course. Obstruction resulting from internal herniation in theory should present late. From the cases described 5 presented within one week of primary operation and the other 5 cases presented 2–9 weeks after primary operation.

## 4. Conclusion

Internal herniation and small bowel obstruction are a rare complication of laparoscopic nephrectomy but should be considered in cases presenting with bowel obstruction early or late in the postoperative course. It is associated with significant morbidity and the need for operative intervention in all reported cases and has been shown to cause ischaemia necessitating bowel resection in one case.

The authors of this report advocate that, during laparoscopic nephrectomy for any purpose, the mesentery must be inspected for defects and any recognised intended or inadvertent defects should be repaired to prevent internal herniation. The hallmarks of best management in cases resulting in obstruction include early CT scanning and surgical intervention with the aim of repairing the mesenteric defect after reduction of the hernia and appropriate management of any bowel ischaemia or perforation. This has been performed laparoscopically in 2 cases; however the decision for open or minimally invasive surgery is dependent on institutional policy and surgeon experience.

## Additional Points

This case report aims to bring attention to a rare complication of laparoscopic nephrectomy and offers recommendations on prevention and management based on literature to date.
